# Amnion as a surrogate tissue reporter of the effects of maternal preeclampsia on the fetus

**DOI:** 10.1186/s13148-016-0234-1

**Published:** 2016-06-10

**Authors:** Masako Suzuki, Ryo Maekawa, Nicole E. Patterson, David M. Reynolds, Brent R. Calder, Sandra E. Reznik, Hye J. Heo, Francine Hughes Einstein, John M. Greally

**Affiliations:** Center for Epigenomics, Department of Genetics, Albert Einstein College of Medicine, 1301 Morris Park Avenue, Bronx, NY 10461 USA; Department of Obstetrics and Gynecology, Yamaguchi University Graduate School of Medicine, Minamikogushi 1-1-1, Ube, 755-8505 Japan; Department of Pharmaceutical Sciences, College of Pharmacy and Health Sciences, St. John’s University, Jamaica, NY 11439 USA; Department of Pathology, Albert Einstein College of Medicine, Bronx, NY 10461 USA; Department of Obstetrics and Gynecology and Women’s Health, Albert Einstein College of Medicine, 1301 Morris Park Avenue, Price 322, Bronx, NY 10461 USA

**Keywords:** Preeclampsia, Hypertension, Pregnancy, DNA methylation, Amnion, Genome-wide, HELP-tagging, Sodium bisulfite

## Abstract

**Background:**

Preeclampsia, traditionally characterized by high blood pressure and proteinuria, is a common pregnancy complication, which affects 2–8 % of all pregnancies. Although children born to women with preeclampsia have a higher risk of hypertension in later life, the mechanism of this increased risk is unknown. DNA methylation is an epigenetic modification that has been studied as a mediator of cellular memory of adverse exposures in utero. Since each cell type in the body has a unique DNA profile, cell subtype composition is a major confounding factor in studies of tissues with heterogeneous cell types. The best way to avoid this confounding effect is by using purified cell types. However, using purified cell types in large cohort translational studies is difficult. The amnion, the inner layer of the fetal membranes of the placenta, is derived from the epiblast and consists of two cell types, which are easy to isolate from the delivered placenta. In this study, we demonstrate the value of using amnion samples for DNA methylation studies, revealing distinctive patterns between fetuses exposed to proteinuria or hypertension and fetuses from normal pregnancies.

**Results:**

We performed a genome-wide DNA methylation analysis, HpaII tiny fragment Enrichment by Ligation-mediated PCR (HELP)-tagging, on 62 amnion samples from the placentas of uncomplicated, normal pregnancies and from those with complications of preeclampsia or hypertension. Using a regression model approach, we found 123, 85, and 99 loci with high-confidence hypertension-associated, proteinuria-associated, and hypertension- and proteinuria-associated DNA methylation changes, respectively. A gene ontology analysis showed DNA methylation changes to be selecting genes with different biological processes in exposure status. We also found that these differentially methylated regions overlap loci previously reported as differentially methylated regions in preeclampsia.

**Conclusions:**

Our findings support prior observations that preeclampsia is associated with changes of DNA methylation near genes that have previously been found to be dysregulated in preeclampsia. We propose that amniotic membranes represent a valuable surrogate fetal tissue on which to perform epigenome-wide association studies of adverse intrauterine conditions.

**Electronic supplementary material:**

The online version of this article (doi:10.1186/s13148-016-0234-1) contains supplementary material, which is available to authorized users.

## Background

Preeclampsia (PE) is a common and potentially serious pregnancy-associated disorder traditionally characterized by de novo maternal hypertension and proteinuria after 20 weeks of gestation [1]. The latest criteria for the definition of PE published by the American Congress of Obstetricians and Gynecologists (ACOG) have changed, with proteinuria being no longer needed for diagnosis, while patients without proteinuria but with new-onset thrombocytopenia, impaired liver function, renal insufficiency, pulmonary edema, or visual or cerebral disturbances are now also diagnosed as having PE [2]. PE, as traditionally defined, affects 2–8 % of all pregnancies and is associated with an increased risk of preterm birth [3]. In recent years, the incidence of PE has risen in the USA [4, 5] and pregnancy-associated hypertension has also become more common [6], which may be attributable to the increased prevalence of predisposing disorders, including chronic hypertension, diabetes, and obesity [7–9]. Not only does PE involve short-term risks to the mother and fetus but children who were exposed to PE in utero have an increased risk of diabetes mellitus (meta-analysis from 16 studies [10]) and higher systolic blood pressure during childhood and young adult life [11–13]. A large population-based cohort study looked at these delayed effects in more detail and showed that children born at term who were exposed to PE were more often hospitalized and had an increased risk of a variety of illnesses, such as endocrine, nutritional, and metabolic diseases throughout childhood and young adult life [14]. These findings suggest that maternal PE somehow causes a detrimental cellular “memory” in the exposed offspring.

The mechanism by which PE causes the exposed fetus to have increased risk of diseases later in life remains unknown [14]. The potential for heritable epigenetic mechanisms to propagate a cellular memory of early life exposures [15–18] makes the molecular mediators of epigenetic regulation strong candidates for mediating the long-term risk to individuals born to mothers with PE, a potentially valuable mechanistic insight. Furthermore, as maternal PE is not deterministic of adult disease in the exposed infants but instead raises relative risk, any potential method to define those individuals at greater risk based on identifying and testing for distinctive epigenetic regulatory marks in accessible cells would be of major value.

Epigenetic memory is believed to be mediated or reflected by chemical modifications of genomic DNA and possibly also chromatin states (reviewed in [19, 20]). DNA methylation is a well-studied transcriptional regulator with heritable epigenetic properties, involving the covalent addition of a methyl group at the 5-carbon position of the cytosine ring. Most DNA methylation occurs at the cytosine of CpG dinucleotides, with 60–80 % of CpG sites methylated in fully differentiated tissues and cells in mammals (reviewed in [21]). During replication, the DNA methylation status is propagated by DNA methyltransferases to daughter chromatids (reviewed in [22]). The changes in DNA methylation that are associated with transcriptional alterations are increasingly recognized to occur at *cis*-regulatory loci that can be distant from gene promoters and are very diversely located in different cell types [23, 24]. As a consequence, genome-wide DNA methylation studies that test as many *cis*-regulatory loci as possible are best positioned to find alterations associated with human diseases [25].

Testing the association between phenotypes and DNA methylation profiles genome-wide (epigenome-wide association studies (EWAS)) is increasingly common [26]. However, EWAS are frequently designed without taking into account major influences on DNA methylation such as genetic variation [27–31], cell subtype composition variation between samples [32, 33], and technical or batch effects [34]. A further problem in human studies is that it is often not possible to access the cell type mediating the disease, instead requiring the use of surrogate tissues or cells. Peripheral blood leukocytes have been used as an accessible surrogate tissue in many EWAS, providing an excellent paradigm for cell subtype variability influencing DNA methylation [33, 35]. To reduce this source of bias in an EWAS, a homogeneous cell type is ideally used, or the application of an analytical approach that allows this source of variability to be addressed [26, 32]. For research into possible epigenetic dysregulation in PE, non-invasive tissue sampling from the neonate exposed to the intrauterine stress is required. If large cohorts of subjects are needed to power these studies [26], it is necessary to define a tissue type that can be easily and uniformly sampled across multiple research groups and institutions.

The amnion is derived from the fetal side of the amniochorionic membrane and shares a common embryological origin with the fetus at the implantation stage. The placental side of the amniochorionic membrane, the chorion, is derived from the trophectoderm. Amnion cells exhibit pluripotent stem cell-like plasticity and can be differentiated into cells expressing markers of mesodermal, endodermal, or ectodermal cells with engraftment or appropriate stimulation by specific differentiation factors [36–38]. The amnion is relatively homogenous in terms of cell composition (mainly epithelial with some mesenchymal stromal cells) and is technically easy to isolate. We hypothesized that these factors make the amnion potentially an excellent, practical surrogate fetal tissue in which to test for epigenetic alterations in neonates.

To test whether epigenetic regulators in the amnion are affected by maternal, we performed genome-wide DNA methylation profiling on 62 amnion samples from infants born to women with PE or hypertension, comparing these with samples from uncomplicated pregnancies, looking for changes recurrently associated with PE exposure. We identified differentially methylated regions between the epithelial and mesenchymal stromal cells of the amnion, allowing us to adjust for the influence of variable cell subtype composition on the DNA methylation patterns observed. Our studies support the value of using the amnion as a focus for studies of adverse intrauterine conditions affecting the fetus.

## Results

### Clinical characteristics of subjects

We show in Table 1 the clinical characteristics of the subjects that we studied, presenting data as mean ± standard deviation (SD) when appropriate. We used a *t* test for continuous variables and Fisher’s exact test for categorical variables when calculating significance of differences between groups. No statistically significant differences between groups for mother’s age at delivery, sex of fetus, and self-reported ancestry were found. The gestational age at delivery was earlier for offspring with complications (PE) compared to those without complications (control) (*p* < 0.05). Although the differences in gestational age were statistically significant, the medians of each group were comparable (39.3, 39.2, and 39.0) with most babies born at term (after 37 weeks). Maternal maximum diastolic and systolic blood pressures were also significantly higher in the pregnancy complication groups compared to controls, as expected (*p* < 0.001). Primiparity, a risk factor for PE (reviewed in [39]), was significantly higher in the PE group (*p* = 0.016). Maternal history of PE, another well-known risk factor for PE, was not significantly different among the groups (*p* = 0.24). Overall, our cohort appears to have the characteristics expected of a typical PE study population.

**Table 1:** Sample characteristics

		Without complication (control)		With complication				
				Hypertension		PE		*p* value
		*N* = 15		*N* = 11		*N* = 36		
Diagnostic criteria	Systolic blood pressure (max)	130	IQR 122–134	160	IQR 148–170	175.5	IQR 157–183	
	Diastolic blood pressure (max)	82	IQR 77–85	98	IQR 93–108	103	IQR 98–115.5	
	Proteinuria							
	Negative/trace	15		11		6		
	1+	0		0		13		
	2+	0		0		8		
	>3+	0		0		9		
Characteristics	Maternal age (year, mean ± SD)	27.3 ± 3.3		26 ± 6.0		26.6 ± 6.1		0.8413
(Mother and fetus)	Week of gestation (median)	39.3	IQR 39.1–40.2	39.2	IQR 37.4–39.5	39	IQR 37.4–39.4	0.0191
	Weight at birth (g, mean ± SD)	3454.3 ± .96		3097.3 ± 606.8		2931.1 ± 767.3		0.0566
	Race							0.58
	Black	4		2		11		
	Hispanic	5		6		19		
	White	1		1		4		
	Other	3		2		1		
	Declined	2		0		1		
	Sex (male, *n*)	5		8		15		0.120
	NSVD (*n*)	5		8		15		0.120
	Primiparous (*n*)	3		6		24		0.016
	Smoked (*n*)	0		0		2		0.999
	History of PE (*n*)	0		2		5		0.242
Comorbidities (*n*)	Anemia	4		1		5		0.502
(Mother)	Asthma	1		3		5		0.419
	Obese (BMI > 30)	4		4		17		0.401
	GDM	1		0		7		0.289
	Chronic hypertension	0		1		4		0.453
	Migraines	1		2		3		0.607
Medication (*n*)	Hypertensive drug prescribed	0		1		9		0.006
(Mother)	MgSO4 administration	0		6		25		<0.0001

*IQR* interquartile range

### Genome-wide DNA methylation profiling in amnion samples

The genome-wide DNA methylation profiles of the 62 amnion samples were evaluated using HpaII tiny fragment Enrichment by Ligation-mediated PCR (HELP)-tagging [40], which offers a better representation of distal *cis*-regulatory elements than other survey assays of DNA methylation [25]. HELP-tagging utilizes the methylation-sensitive restriction enzyme HpaII and its methylation-insensitive isoschizomer MspI to test the methylation status at HpaII sites, generating an angular metric that results in accurate quantification of DNA methylation at >1.8 million loci in the human genome [40]. Technical verification was performed with bisulfite amplicon-seq using the Fluidigm micro-fluidic amplification system (mean Pearson correlation 0.78, interquartile range −0.84 to −0.78; see Additional file 1 for detailed methods, analysis, and graphical data representations). This verification confirmed that the genome-wide data were robust enough to allow interpretation. There are several known confounding influences on genome-wide DNA methylation assays, including technical/batch effects, DNA sequence variants, cell subtype composition variability, and sex/age [26, 27]. We addressed technical effects (batch effects) using ComBat, keeping group information (PE, hypertension, and control) as the outcome of interest (see Additional file 1 for analysis). We addressed the possibility of local DNA sequence polymorphism by eliminating loci from analysis where the ±28 bp flanking the HpaII site overlaps a known single nucleotide polymorphism (SNP, dbSNP142). We further eliminated from analysis HpaII sites where the MspI control tag count was low (<4), or located on the sex chromosomes or at a repetitive element (RepeatMasker annotation http://www.repeatmasker.org/). These conservative measures left us with 654,051 HpaII sites for analysis.

### Eliminating cell subtype composition effects

Although the amnion is less heterogeneous in terms of cell subtypes than the other parts of the placenta, it consists of two morphologically distinct cell types, amniotic epithelial (AE) and amniotic stromal (AS) cells. We purified these two cell types from amnion samples from four individuals and performed whole-genome bisulfite sequencing of each of the ten samples (including replicates), identifying differentially methylated regions using Fisher’s exact test [41]. As we expected, DNA methylation profiles showed cell type-specific patterns. We identified three strongly differentially methylated HpaII sites, Hpa_1553647, Hpa_210409, and Hpa_621984 (Additional file 2: Figure S1). Hpa_1553647 is located in the intron of the signal-induced proliferation-associated 1-like 1 (*SIPA1L1*) gene. *SIPA1L1* encodes a protein that stimulates the Rap GTPase activity [42] and has been identified as a human papillomavirus E6 targeted protein [43]. As there is no transcript reported within 50 kb of Hpa_210409, we are unable to speculate about the function of this locus. Hpa_621984 is located in the intron of polo-like kinase 2 (*PLK2*) gene, which is associated with cell cycle functions [44]. We hypothesized that DNA methylation variability at these differentially methylated sites could be associated with variability at multiple sites across samples, reflecting differences in proportions of AE to AS cells in different samples. We also tested whether any of these loci had DNA methylation changes associated with the clinical phenotypes being studied. We found Hpa_621984 to have only a weak association with DNA methylation variability (*p* = 0.866, Additional file 3: Table S1) and a strong association between Hpa_210409 and proteinuria (*p* = 0.008892). The remaining HpaII_1553647 locus was therefore used as the most robust indicator of cell subtype proportions. We removed all HpaII sites which showed strong correlations with HpaII_1553647 (*r* > 0.3, Spearman’s correlation). This left 545,961 HpaII sites to test the DNA methylation changes specific to PE exposure.

**Fig. 1:**
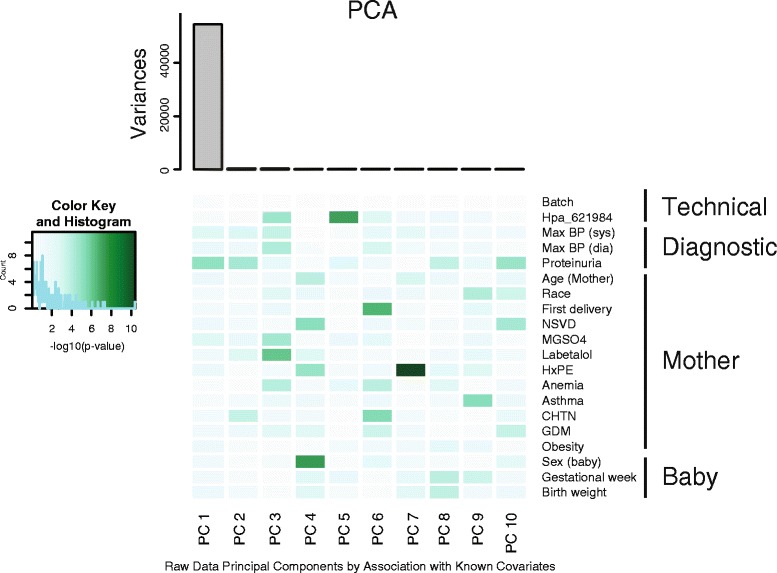
Biological and technical confounders contribute to DNA methylation value variations. The heatmap displays significant correlations for each covariate. The –log_10_ *p* values of the linear regressions of the top ten principal components onto each known covariate are shown. The *color key* shows corresponding numeric values, with *darker color* indicating increased significance. Proteinuria, maximum systolic blood pressure, and magnesium sulfate treatment are contributing to the DNA methylation variability

Using this high-confidence data set, we tested the degree of contribution of known clinical covariates to DNA methylation profiles using principal components analysis (PCA, Fig. 1). We observed a significant contribution of proteinuria (*p* = 0.0305) and a strong contribution of maximum systolic blood pressure and magnesium sulfate treatment (*p* = 0.157 and 0.160, respectively) to the DNA methylation variability in our study. Magnesium sulfate has been used for preventing seizures in women with PE. These results suggested that there are PE exposure-specific DNA methylation changes in the amnion.

### Transcriptional studies of the amnion reveal expression level changes of genes previously implicated in PE

Transcription and DNA methylation are potentially associated [45–47], prompting our exploration of this relationship in our study. We performed directional RNA-seq on 12 without complication (control) and 17 PE amnion samples (Additional file 1, detailed method with technical verification and analysis). We found 41 high-confidence differentially expressed genes between the PE and control amnions, a list including genes previously associated with PE (*fms*-*like tyrosine kinase 1* (*FLT1*) [48, 49], *S100 calcium binding protein A8*/*A9* (*S100A8*/*A9*) [50, 51], *pregnancy-associated plasma protein A2* (*PAPPA2*) [52], and *C-X-C motif chemokine* (*CXCL8*) [53, 54] (Additional file 3: Table S2, Additional file 4: Figure S2). Soluble FLT1 (sFLT1) is a well-known anti-angiogenic factor which has been found to increase in expression in PE (reviewed in [39]) with animal models indicating that its over-expression leads to PE-like symptoms [48]. *S100A8* and *CXCL8* are known inflammation-related genes; S100A8 has been reported to have increased expression levels in amnion samples from mothers with PE and inflammation [55]. Up-regulation of *CXCL8* was also reported to be part of the response to pro-inflammatory cytokines in first trimester decidual cells [56]. Macintire et al. reported that *PAPPA2* expression is increased in severe early-onset PE and is upregulated with hypoxia [52]. Both inflammation and hypoxia are known contributors to PE [57–61]. We did not, however, observe local DNA methylation alterations in proximity to these differentially expressed genes in our study (Additional file 4: Figure S2), suggesting either that DNA methylation is not involved in the regulation of these genes or that the regulation occurs at CG dinucleotides that were not tested using HELP-tagging.

### Defining loci of differential DNA methylation attributable to PE exposure

**Fig. 2:**
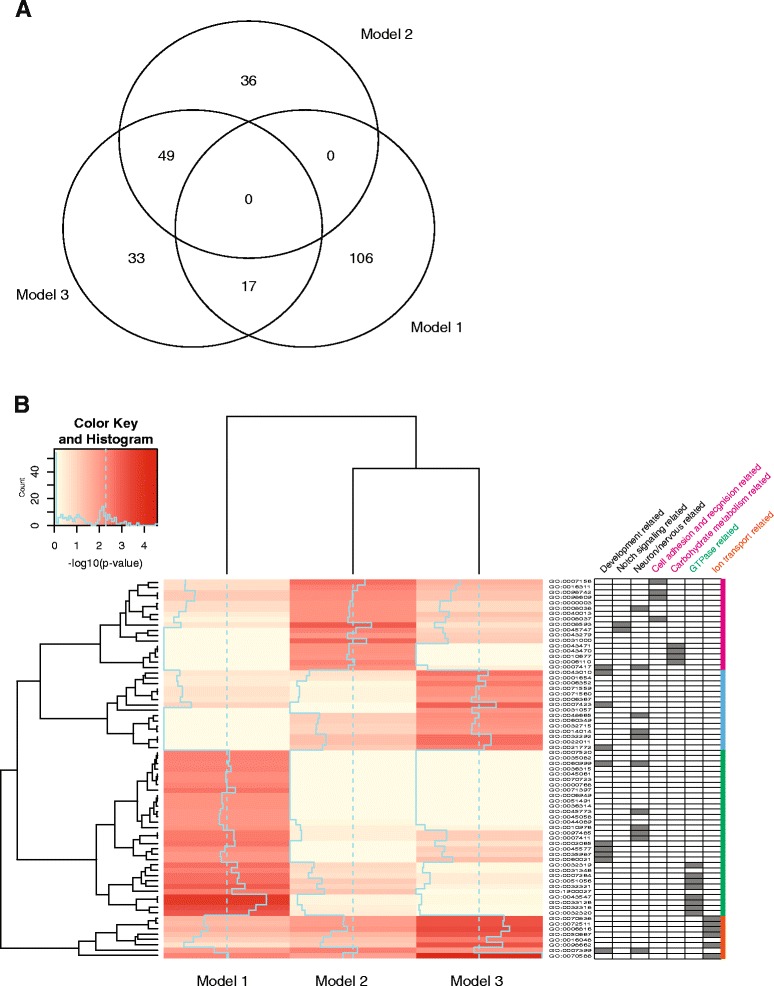
Differentially methylated HpaII (DM-HpaII) sites. **a** DM-HpaIIs common in between models, a Venn diagram showing the overlapping DM-HpaIIs between models. **b** Exposures of maternal hypertension and proteinuria showed different target genes and biological process. The heatmap displays the –log_10_ *p* values of gene ontology enrichment status (Enrichr, GO biological process). Distinct patterns of enrichment for gene ontology biological processes between models were observed

To identify DNA methylation changes associated with PE exposure, we used a linear modeling approach. The PCA results suggested that maximum systolic blood pressure and proteinuria grade were strong contributors to DNA methylation changes. We therefore ran three regression models and measured the proportion of variance explained from each: model 1, testing the effect of maximum systolic blood pressure; model 2, the effect of proteinuria grade; and model 3, the combination of both. We used ANOVA to test the significance of the DNA methylation changes in each model. We further applied a stringent threshold (false discovery rate (FDR)-adjusted *p* values of <0.05 and differences between PE and control >10) and identified 123, 85, and 99 high-confidence differentially methylated HpaII (DM-HpaII) sites for each of the three models, respectively (Additional file 3: Table S3). In Fig. 2a, we show the overlap of DM-HpaII sites between the models. As would be expected, we found DM-HpaII sites to overlap between the combined model 3 and the individual models 1 and 2. Gene ontology analysis showed distinct patterns of enrichment for biological processes between models. Cell-cell adhesion- and recognition-related and carbohydrate metabolic-related genes were enriched in model 2, while GTP-associated genes were enriched in model 1 (Fig. 2b). This suggests that exposures of maternal hypertension and proteinuria have different target genes and biological consequences.

There are several DNA methylation analysis studies on PE patients. A search of the PubMed database with the terms “preeclampsia”/“pre-eclampsia,” “DNA methylation,” and “genome-wide”/“genome wide” finds 12 publications [62–73], of which two entries did not perform genome-wide DNA methylation studies and one did not study PE. Most studies were focused on the etiology of PE; hence, most of the studies were performed on trophectoderm-derived (placenta) or maternal blood cells or a trophectoderm and epiblast mixed population (chorionic membrane). One study used an epiblast-derived tissue (cord blood cells) [63] and reported a list of 319 genes as PE and gestational age-associated, differentially methylated genes using Illumina Infinium HumanMethylation450 BeadChip arrays. Because of the platform difference, we are not able to compare systematically the individual CpG sites. A small number (*GABBR1*, *HMHA*, *TSPAN18*, *CACNA2D3*, and *SKI*) of the 319 genes were also found to be differentially methylated in our current study.

Seven DM-HpaIIs overlapped probes of the Illumina Infinium HumanMethylation450 BeadChip. Of these, six have been associated with aging [74, 75], obesity [76], osteoarthritis [77], and liver development [78] (Additional file 3: Table S4). A CpG site (Hpa_1775280/cg04521626) was reported to be differentially methylated in two different studies using cord blood samples studying birth weight and gestational age [79, 80]. Although Hpa_1775280/cg04521626 did not show significant association on our regression models, we observed significant associations between DNA methylation at this locus with birth weight and with week of gestation (*p* = 0.00814 and *p* = 0.00046, respectively).

Preeclampsia (PE) can be categorized based on the time of onset, as early onset (EOPE) or late onset (LOPE). While some clinical features overlap between EOPE and LOPE, it has been reported that the etiologies of disease, biomarkers, and maternal and fetus outcome are different between EOPE and LOPE (reviewed in [81]). Furthermore, the severities of hypertension and proteinuria differ between patients. This suggests that the degree of DNA methylation alterations may also vary among the PE-exposed fetuses. Therefore, we searched for CpG sites where DNA methylation variation within groups is significantly higher in the PE-exposed than that in control fetuses. We compared the variances of each HpaII site in the controls and PE-exposed fetuses with the F test (R project, stats package) and found 4058 variable HpaII (var-HpaII) sites in 3035 genes between the control and PE (FDR-adjusted *p* value <10^−7^ and the ratio of variance >20) (Additional file 3: Table S5). Of those, 28 genes had more than 5 var-HpaII sites, and several of those have been reported the association with PE (*PTPRN2*, *KCNMA1*, and *NFATC1*), hypertension (*SDK1*), and placental development (*SALL3*) (Additional file 3: Table S6). We verified the increased variability of DNA methylation predicted by HELP-tagging results using bisulfite MassArray (Additional file 5: Figure S3 and Additional file 6: Figure S4).

## Discussion

While the amnion is not part of the fetal soma and cannot mediate the long-term PE effects on disease risk, our hypothesis was that it provides an easily accessible surrogate cell type reflecting fetal cell exposure to the intrauterine stress of PE. The effects of PE on fetal cells are not deterministic but instead influence the relative risk of developing long-term disease phenotypes. In this study, we showed that PE exposure-associated differentially and variably methylated genes overlap with previously reported PE-associated differentially methylated genes in studies of other tissue types. Our results support the use of the amnion as surrogate tissue in studies of PE and other adverse intrauterine conditions. The amnion membrane consists only two cell types: AE and AS cells. Since each cell type has a unique DNA methylation profile, variability in cell subtype composition between samples can be a strong confounding factor when testing DNA methylation variability between samples. Our identification of differentially methylated loci between the two cell types of the amnion allows us to apply an approach similar to Houseman et al. [82] to adjust for cell subtype proportional variability, increasing the interpretability of data derived from studies of the amnion. The amnion is therefore a promising surrogate tissue for studies of PE and potentially other intrauterine stresses, as it is easy to collect, composed of only two cell types whose proportions can be reported by differentially methylated regions that we have identified, and shows DNA methylation changes similar to those found in other studies of cells of fetuses exposed to PE.

This study is the largest to date in terms of numbers of individuals studied using genome-wide DNA methylation assays in PE (62 samples in total). Despite this, we recognize that a larger cohort would be needed to detect smaller changes in DNA methylation with confidence, and we have not been able to account for the effects of DNA sequence variants on DNA methylation, methylation quantitative trait loci (meQTLs) [30, 31, 83, 84]. Furthermore, while we adjusted for epithelial/stromal proportions, we only used a single high-confidence DMR for this analysis. Additionally, although we collected samples from the placenta whose mother was not diagnosed with chorioamnionitis or other inflammatory disorder in this study, we cannot ignore the possibility of occasional maternal cell contamination. Finally, the ~2 million loci tested using HELP-tagging only represent a subset of CpG dinucleotides in the human genome, which is likely to miss the majority of distal *cis*-regulatory elements [25]. A future, more definitive study would have to be more comprehensive, adding genotyping and cell types and using a larger cohort. A focus on the amnion would facilitate such an expanded study as it is very easy to dissect from the delivered placenta, facilitating a standardized sample collection in a multicenter project. The results of the current study indicate that the amnion could potentially be the focus of research performed by diverse groups interested in the effects of adverse intrauterine conditions, allowing integration of information obtained among different investigators.

In this study, we identified 123, 85, and 99 high-confidence differentially methylated HpaII (DM-HpaII) sites for hypertension, proteinuria, and hypertension-proteinuria regression models, respectively, and 4058 variable HpaII (var-HpaII) sites. These differences persisted despite rigorous measures to account for variability in cell subtype composition, potential DNA sequence variation, and technical artifacts, with the technical performance of the genome-wide assay verified with a highly quantitative and orthogonal assay. A gene ontology analysis showed DNA methylation (DM-HpaII) changes to be selecting genes with different biological processes in exposure status. Cell-cell adhesion- and recognition-related and carbohydrate metabolic-related genes were enriched in model 2 (proteinuria), while GTPase-associated genes were enriched in model 1 (hypertension). This suggests a model of exposures to maternal hypertension and maternal proteinuria affecting different target genes, with distinct biological consequences. The associations between PE exposure and diabetes later in life have been identified in a couple of large cohort studies [14, 85]. In addition, many molecular and cellular studies have identified the roles of GTPase in the regulation of vascular smooth muscle cell proliferation and migration (reviewed in [86]). While offspring outcomes after birth are not available from our cohort, these alterations might conceivably be associated with diseases later in life. It has been demonstrated that *NFATC1* induces COX-2 expression in a rat model [87]. Induction of COX-2 expression has been reported in syncytiotrophoblast cells and systemic vascular tissue of PE patients [88, 89], and COX-2 expression has been related to reduced blood flow in PE patients [89]. A mouse model study reported that the *SALL3* locus is a hotspot of epigenetic alterations associated with placentomegaly of cloned mice. They reported that the DNA methylation status at the *SALL3* locus was associated with placental sizes of the animal [90]. It has been shown that the size of the placenta in EOPE tends to small and that this size is associated with the baby’s weight, while a U-shaped placental size distribution was reported in pregnancies with LOPE [91]. It has been reported that low placental weight at birth is associated with an increased risk of hypertension of the offspring later in life [11, 13]. While further longitudinal studies are needed to test the associations between epigenetic alterations in this locus and the disease prevalence of the offspring, the epigenetic signature at the *SALL3* locus might be a potential risk predictor of later diseases of the offspring.

## Conclusions

To our knowledge, this is the largest study to address genome-wide DNA methylation changes in PE-exposed babies with adjustment for cell composition variations. Our findings support previous evidence that exposure to PE changes methylation of the fetal DNA. The differentially methylated genes and associated pathways we identified here are in line with those found in previous studies testing the effect of PE on fetal epigenetic programming. We propose amniotic membranes as the best surrogate tissue to perform large-scale, epigenome-wide association studies of intrauterine stresses.

## Methods

### Sample collection

Amnion samples were collected immediately after delivery from women with and without PE at Montefiore Medical Center, the university hospital of the Albert Einstein College of Medicine. To eliminate maternal blood contamination, we gently washed the collected membrane twice with 0.9 % NaCl and once with RNAlater solution. Then the washed membrane samples were temporarily stored in the RNAlater solution to protect RNAs from nucleases, at 4 °C for up to 20 h, and then the samples were cut into small pieces and stored at −80 °C until use. Diagnosis of PE was defined as follows: sustained systolic blood pressure of ≥140 mmHg or sustained diastolic blood pressure of ≥90 mmHg on 2 separate readings; proteinuria measurement of 1+ or more on a dipstick; or a 24-h urine protein collection with 300 mg in specimen or observed other PE clinical characteristics. Women who did not have proteinuria or other PE clinical characteristics but had increased blood pressure (sustained systolic blood pressure of ≥140 mmHg or sustained diastolic blood pressure of ≥90 mmHg on two separate readings) were grouped as having with complication (hypertension). This study was approved by the Institutional Review Board (IRB) for Human Research at Albert Einstein College of Medicine, Bronx, NY.

### Cell isolation and culture

To isolate fresh cells, the collected amnions or umbilical cords were washed in 100 U/ml penicillin and 100 μg/ml streptomycin-supplemented Dulbecco’s modified Eagle’s medium (DMEM) at room temperature followed by immediate cell isolation. For amniotic epithelial (AE) and amniotic stromal (AS) cells, we isolated five pairs of AE and AS cells from the same individuals with series of digestions of amnion membrane [92]. Isolated cells were snap-frozen or maintained in a 1:1 mixture of Ham’s F-12 and DMEM supplemented with 10 % fetal bovine serum (FBS), 100 U/ml penicillin, and 100 μg/ml streptomycin.

### DNA and RNA extraction

DNA samples were extracted with a standard phenol-chloroform treatment method [40], and total RNA samples were extracted with TRIzol reagent.

### Genome-wide DNA methylation assays

We evaluated genome-wide DNA methylation profiles in amnion samples with our previously published optimized tag-based DNA methylation assay (HELP-tagging) [40]. One microgram of each sample was digested with HpaII as a starting material. We used deeply sequenced MspI libraries generated from commercially available pooled human genomic DNA (Roche) for DNA methylation calculation. The degree of methylation was calculated using our previously reported angle transformation method [40]. The results were verified using a bisulfite treatment based micro-methylation-seq method (detailed protocol and results shown in Additional file 7). To identify a cell type-specific DNA methylation, we used low-coverage whole-genome bisulfite sequencing, selecting differentially methylated loci using Fisher’s exact test (*p* < 0.00001). The obtained candidate HpaII sites were verified using conventional bisulfite sequencing or bisulfite MassArray.

### Single locus DNA methylation assays

We performed single locus DNA methylation assays to assess the DNA methylation status of candidate HpaII sites we identified with genome-wide DNA methylation assays using bisulfite MassArray [93]. We designed bisulfite primers using MethPrimer (http://www.urogene.org/methprimer/), testing the primer’s specificities with BiSearch (http://bisearch.enzim.hu/) and the amplicon’s analyzability with the amplicon prediction function of MassArray [93] (Bioconductor, http://www.bioconductor.org/packages/release/bioc/html/MassArray.html). The primer sequences used in this study are listed in Additional file 8. Five hundred nanograms of genomic DNA was treated with sodium bisulfite using the EZ DNA Methylation Gold Kit (Zymo Research). Bisulfite-treated DNA samples were used as a template for PCR amplification using the following condition: 2.5 μl of 10x PCR buffer, 1 μl of 20 μM each primer, 0.5 μl of 10 mM dNTP mix, FastStart HiFi polymerase, and 10 ng of template (as starting material) in a final volume of 25 μl; 95 °C for 10 min, then 42 cycles of 95 °C for 30 s, annealing temperatures for 30 s and 74 °C for 30 s followed by 10 min at 74 °C for the final extension.

### Directional RNA-seq

The transcription profiles were defined using directional RNA-seq. We used 500 ng of total RNA as starting material. Before reverse transcription, the ribosomal RNAs were depleted with Ribo-Zero rRNA removal kit (Epicentre). The reverse transcription was performed using the SuperScript III First-Strand Synthesis System followed by second strand cDNA synthesis with deoxyuridine triphosphates (dUTPs). Synthesized double-stranded cDNAs were fragmented with Covaris (200–300 bp as target length), end-repaired, dA tailed and adapters added for the Illumina sequencer. To maintain the directional information, we used a combination of dUTP incorporation and uracil-DNA glycosylase [94]. All libraries were quality-checked before the sequence and sequenced on Illumina HiSeq 2500 (100 bp single-end reads). The verification of directional RNA-seq was performed with quantitative RT-PCR. The detailed protocol and the verification results are shown in Additional file 7.

### Bioinformatic analyses

All bioinformatics analyses we used here were performed on a high-performance computing cluster of Albert Einstein College of Medicine. We used the following packages: FASTQC, Trim_Galore (http://www.bioinformatics.babraham.ac.uk/projects/trim_galore/), bismark [95], samtools [96], bedtools (http://bedtools.readthedocs.org/en/latest/#), STAR [97], HTseq-count [98], and R version 3.2.1 (http://www.r-project.org/, 3.2.1) with DEseq2 [99], NOIseq [100], gplot (https://cran.r-project.org/web/packages/gplots/index.html), sva (http://bioconductor.org/packages/release/bioc/html/sva.html), and stats.

All the codes used in this study are listed in Additional file 7 and are publicly available at our GitHub server: https://github.com/GreallyLab/PE_Suzuki_et_al_2016.

## Abbreviations

cDNA, complementary DNA; CpG, cytosine-phosphate-guanine; DM-HpaII, differentially methylated HpaII; DNA, deoxyribonucleic acid; dUTP, deoxyuridine triphosphate; EOPE, early onset preeclampsia; FDR, false discovery rate; HELP, HpaII tiny fragment Enrichment by Ligation-mediated PCR; IQR, interquartile range; LOPE, late onset preeclampsia; PE, preeclampsia; rRNA, ribosomal ribonucleic acid; RT-PCR, reverse transcription polymerase chain reaction; var-HpaII, variably methylated HpaII

## Additional files

Additional file 1: We described the study design, detailed analytical methods, and verification results in the supporting information file. (DOCX 21.2 MB) Additional file 2: Figure S1. Cell type-specific DNA methylation. A) Bisulfite sequencing results of Hpa_1553647. Each row represents the sequence result from bisulfite PCR products. We show four amnion, four amniotic epithelial cell, and amniotic stromal cell results. The amniotic epithelial and stromal cells were isolated from four individuals. The blue arrow indicates the Hpa_1553647 position in the sequencing results. B) Bisulfite MassArray results of Hpa_210409 and Hpa_621984. The *y*-axis shows the % DNA methylation in amniotic epithelial and amniotic stromal cells (from four individuals). The *p* values were calculated by a *t* test. The error bars indicate the standard deviations. (PDF 317 kb) Additional file 3: Table S1. Lists of *p* values of principal component analysis with known covariates. **Table S2.** A list of differentially expressed genes. **Table S3.** Lists of differentially methylated HpaII sites in each model. **Table S4.** A list of differentially methylated HpaII sites with previously reported Infinium HumanMethylation450 BeadChip differentially methylated probes. **Table S5.** A list of variably methylated HpaIIs. **Table S6.** A list of var-HpaII sites harboring genes. (XLSX 374 kb) Additional file 4: Figure S2. Differentially expressed genes in preeclampsia-exposed amnion. A MA plot shows the distribution of differentially expressed and differentially methylated genes. The *x*-axis shows counts per million (CPM), and the *y*-axis shows log-fold changes. The differentially methylated genes are highlighted in red and the differentially expressed genes are highlighted in green. (PDF 4.30 MB) Additional file 5: Figure S3. Genes with local variable HpaII sites. HpaII sites with variable DNA methylation are shown at specific loci with HELP-tagging (angle values = (1 − DNA methylation value)) and corresponding bisulfite MassArray verification results. The var-HpaII track indicates loci that could be analyzed by bisulfite MassArray in red, and those that could not be analyzed in gray (PCR amplification problem or more than 2 CpG sites in a MassArray fragment). The blue loci are those in which the analysis of the amplicon indicated the likely presence of a sequence polymorphism [93]. Top panel: *SALL3* region, bottom panel: *KCNMA1* region. (PDF 1.33 MB) Additional file 6: Figure S4. Comparison of the DNA methylation distribution of variable HpaII sites. The distributions of the DNA methylation levels of variable methylation sites in severe PE-exposed (PE_S, green) (proteinuria grade ≥3 and systolic blood pressure ≥160 mmHg), less severe PE-exposed (PE_M, orange) (proteinuria grade ≤1 and systolic blood pressure ≥ 140 mmHg) and control (blue) were summarized graphically in violin plots. (PDF 895 kb) Additional file 7: Codes used in this study. (TXT 66.7 kb) Additional file 8: Supporting Tables. (XLSX 15.7 MB)

## References

[1] ACOG practice bulletin (2002). Diagnosis and management of preeclampsia and eclampsia. Number 33, January 2002. Obstet Gynecol.

[2] American College of O, Gynecologists, Task Force on Hypertension in P (2013). Hypertension in pregnancy. Report of the American College of Obstetricians and Gynecologists’ Task Force on Hypertension in Pregnancy. Obstet Gynecol.

[3] Ghulmiyyah L, Sibai B (2012). Maternal mortality from preeclampsia/eclampsia. Semin Perinatol.

[4] Steegers EA, von Dadelszen P, Duvekot JJ, Pijnenborg R (2010). Pre-eclampsia. Lancet.

[5] Wallis AB, Saftlas AF, Hsia J, Atrash HK (2008). Secular trends in the rates of preeclampsia, eclampsia, and gestational hypertension, United States, 1987–2004. Am J Hypertens.

[6] Martin JH, Hamilton BE, Sutton PD, Ventura SJ, Menacker F, Kirmeyer S, Mathews TJ, Statistics DoV. Statistics DoV: births: final data for 2006. (SERVICES USDOHH ed., vol. 57. Hyattsville: National Center for Health Statistics; 2009.

[7] Ogden CL, Lamb MM, Carroll MD, Flegal KM. Obesity and socioeconomic status in adults: United States 1988–1994 and 2005–2008. NCHS data brief no 50. Hyattsville, MD: National Center for Health Statistics; 2010. http://www.cdc.gov/nchs/products/databriefs/db50.htm. 21211165

[8] Fang J, Ayala C, Loustalot F, Dai S (2013). Self-reported hypertension and use of antihypertensive medication among adults—United States, 2005–2009. MMWR.

[9] Geiss LS, Li Y, Kirtland K, Barker L, Burrows NR, Gregg EW (2012). Increasing prevalence of diagnosed diabetes—United States and Puerto Rico, 1995–2010. MMWR.

[10] Henry EB, Patterson CC, Cardwell CR (2011). A meta-analysis of the association between pre-eclampsia and childhood-onset type 1 diabetes mellitus. Diabet Med.

[11] Davis EF, Lazdam M, Lewandowski AJ, Worton SA, Kelly B, Kenworthy Y, Adwani S, Wilkinson AR, McCormick K, Sargent I (2012). Cardiovascular risk factors in children and young adults born to preeclamptic pregnancies: a systematic review. Pediatrics.

[12] Kajantie E, Eriksson JG, Osmond C, Thornburg K, Barker DJ (2009). Pre-eclampsia is associated with increased risk of stroke in the adult offspring: the Helsinki birth cohort study. Stroke.

[13] Tenhola S, Rahiala E, Martikainen A, Halonen P, Voutilainen R (2003). Blood pressure, serum lipids, fasting insulin, and adrenal hormones in 12-year-old children born with maternal preeclampsia. J Clin Endocrinol Metab.

[14] Wu CS, Nohr EA, Bech BH, Vestergaard M, Catov JM, Olsen J (2009). Health of children born to mothers who had preeclampsia: a population-based cohort study. Am J Obstet Gynecol.

[15] Hon GC, Rajagopal N, Shen Y, McCleary DF, Yue F, Dang MD, Ren B (2013). Epigenetic memory at embryonic enhancers identified in DNA methylation maps from adult mouse tissues. Nat Genet.

[16] Thompson RF, Fazzari MJ, Niu H, Barzilai N, Simmons RA, Greally JM (2010). Experimental intrauterine growth restriction induces alterations in DNA methylation and gene expression in pancreatic islets of rats. J Biol Chem.

[17] Ozanne SE, Constancia M (2007). Mechanisms of disease: the developmental origins of disease and the role of the epigenotype. Nat Clin Pract Endocrinol Metab.

[18] Wu G, Bazer FW, Cudd TA, Meininger CJ, Spencer TE (2004). Maternal nutrition and fetal development. J Nutr.

[19] Bird A (2002). DNA methylation patterns and epigenetic memory. Genes Dev.

[20] Levenson JM, Sweatt JD (2005). Epigenetic mechanisms in memory formation. Nat Rev Neurosci.

[21] Smith ZD, Meissner A (2013). DNA methylation: roles in mammalian development. Nat Rev Genet.

[22] Jones PA, Liang G (2009). Rethinking how DNA methylation patterns are maintained. Nat Rev Genet.

[23] Meissner A, Mikkelsen TS, Gu H, Wernig M, Hanna J, Sivachenko A, Zhang X, Bernstein BE, Nusbaum C, Jaffe DB (2008). Genome-scale DNA methylation maps of pluripotent and differentiated cells. Nature.

[24] Shiota K, Kogo Y, Ohgane J, Imamura T, Urano A, Nishino K, Tanaka S, Hattori N (2002). Epigenetic marks by DNA methylation specific to stem, germ and somatic cells in mice. Genes Cells.

[25] Ulahannan N, Greally JM (2015). Genome-wide assays that identify and quantify modified cytosines in human disease studies. Epigenetics Chromatin.

[26] Michels KB, Binder AM, Dedeurwaerder S, Epstein CB, Greally JM, Gut I, Houseman EA, Izzi B, Kelsey KT, Meissner A (2013). Recommendations for the design and analysis of epigenome-wide association studies. Nat Methods.

[27] Berko ER, Suzuki M, Beren F, Lemetre C, Alaimo CM, Calder RB, Ballaban-Gil K, Gounder B, Kampf K, Kirschen J (2014). Mosaic epigenetic dysregulation of ectodermal cells in autism spectrum disorder. PLoS Genet.

[28] Banovich NE, Lan X, McVicker G, van de Geijn B, Degner JF, Blischak JD, Roux J, Pritchard JK, Gilad Y (2014). Methylation QTLs are associated with coordinated changes in transcription factor binding, histone modifications, and gene expression levels. PLoS Genet.

[29] Smith AK, Kilaru V, Kocak M, Almli LM, Mercer KB, Ressler KJ, ylavsky FA, Conneely KN. Methylation quantitative trait loci (meQTLs) are consistently detected across ancestry, developmental stage, and tissue type. BMC Genomics. 2014;15:145. 10.1186/1471-2164-15-145 PMC4028873 24555763

[30] Bell JT, Pai AA, Pickrell JK, Gaffney DJ, Pique-Regi R, Degner JF, Gilad Y, Pritchard JK (2011). DNA methylation patterns associate with genetic and gene expression variation in HapMap cell lines. Genome Biol.

[31] Gibbs JR, van der Brug MP, Hernandez DG, Traynor BJ, Nalls MA, Lai SL, Arepalli S, Dillman A, Rafferty IP, Troncoso J (2010). Abundant quantitative trait loci exist for DNA methylation and gene expression in human brain. PLoS Genet.

[32] Houseman EA, Christensen BC, Yeh RF, Marsit CJ, Karagas MR, Wrensch M, Nelson HH, Wiemels J, Zheng S, Wiencke JK, Kelsey KT (2008). Model-based clustering of DNA methylation array data: a recursive-partitioning algorithm for high-dimensional data arising as a mixture of beta distributions. BMC Bioinformatics.

[33] Lam LL, Emberly E, Fraser HB, Neumann SM, Chen E, Miller GE, Kobor MS (2012). Factors underlying variable DNA methylation in a human community cohort. Proc Natl Acad Sci U S A.

[34] Johnson WE, Li C, Rabinovic A (2007). Adjusting batch effects in microarray expression data using empirical Bayes methods. Biostatistics.

[35] Koestler DC, Christensen B, Karagas MR, Marsit CJ, Langevin SM, Kelsey KT, Wiencke JK, Houseman EA (2013). Blood-based profiles of DNA methylation predict the underlying distribution of cell types: a validation analysis. Epigenetics.

[36] Wei JP, Zhang TS, Kawa S, Aizawa T, Ota M, Akaike T, Kato K, Konishi I, Nikaido T (2003). Human amnion-isolated cells normalize blood glucose in streptozotocin-induced diabetic mice. Cell Transplant.

[37] Ilancheran S, Michalska A, Peh G, Wallace EM, Pera M, Manuelpillai U (2007). Stem cells derived from human fetal membranes display multilineage differentiation potential. Biol Reprod.

[38] Bailo M, Soncini M, Vertua E, Signoroni PB, Sanzone S, Lombardi G, Arienti D, Calamani F, Zatti D, Paul P (2004). Engraftment potential of human amnion and chorion cells derived from term placenta. Transplantation.

[39] Redman CW, Sargent IL (2005). Latest advances in understanding preeclampsia. Science.

[40] Suzuki M, Jing Q, Lia D, Pascual M, McLellan A, Greally JM (2010). Optimized design and data analysis of tag-based cytosine methylation assays. Genome Biol.

[41] Hansen KD, Langmead B, Irizarry RA (2012). BSmooth: from whole genome bisulfite sequencing reads to differentially methylated regions. Genome Biol.

[42] Tsai IC, Amack JD, Gao ZH, Band V, Yost HJ, Virshup DM (2007). A Wnt-CKIvarepsilon-Rap1 pathway regulates gastrulation by modulating SIPA1L1, a Rap GTPase activating protein. Dev Cell.

[43] Gao Q, Srinivasan S, Boyer SN, Wazer DE, Band V (1999). The E6 oncoproteins of high-risk papillomaviruses bind to a novel putative GAP protein, E6TP1, and target it for degradation. Mol Cell Biol.

[44] Ma S, Charron J, Erikson RL (2003). Role of Plk2 (Snk) in mouse development and cell proliferation. Mol Cell Biol.

[45] Zhang X, Yazaki J, Sundaresan A, Cokus S, Chan SW, Chen H, Henderson IR, Shinn P, Pellegrini M, Jacobsen SE, Ecker JR (2006). Genome-wide high-resolution mapping and functional analysis of DNA methylation in Arabidopsis. Cell.

[46] Zilberman D, Gehring M, Tran RK, Ballinger T, Henikoff S (2007). Genome-wide analysis of Arabidopsis thaliana DNA methylation uncovers an interdependence between methylation and transcription. Nat Genet.

[47] Ball MP, Li JB, Gao Y, Lee JH, LeProust EM, Park IH, Xie B, Daley GQ, Church GM (2009). Targeted and genome-scale strategies reveal gene-body methylation signatures in human cells. Nat Biotechnol.

[48] Lu F, Longo M, Tamayo E, Maner W, Al-Hendy A, Anderson GD, Hankins GD, Saade GR (2007). The effect of over-expression of sFlt-1 on blood pressure and the occurrence of other manifestations of preeclampsia in unrestrained conscious pregnant mice. Am J Obstet Gynecol.

[49] Levine RJ, Maynard SE, Qian C, Lim KH, England LJ, Yu KF, Schisterman EF, Thadhani R, Sachs BP, Epstein FH (2004). Circulating angiogenic factors and the risk of preeclampsia. N Engl J Med.

[50] Braekke K, Holthe MR, Harsem NK, Fagerhol MK, Staff AC (2005). Calprotectin, a marker of inflammation, is elevated in the maternal but not in the fetal circulation in preeclampsia. Am J Obstet Gynecol.

[51] Founds SA, Terhorst LA, Conrad KP, Hogge WA, Jeyabalan A, Conley YP (2011). Gene expression in first trimester preeclampsia placenta. Biol Res Nurs.

[52] Macintire K, Tuohey L, Ye L, Palmer K, Gantier M, Tong S, Kaitu'u-Lino TJ. PAPPA2 is increased in severe early onset pre-eclampsia and upregulated with hypoxia. Reprod Fertil Dev. 2014;26:351–7. 10.1071/RD12384 23484525

[53] Sharma A, Satyam A, Sharma JB (2007). Leptin, IL-10 and inflammatory markers (TNF-alpha, IL-6 and IL-8) in pre-eclamptic, normotensive pregnant and healthy non-pregnant women. Am J Reprod Immunol.

[54] Velzing-Aarts FV, Muskiet FA, van der Dijs FP, Duits AJ (2002). High serum interleukin-8 levels in Afro-Caribbean women with pre-eclampsia. Relations with tumor necrosis factor-alpha, Duffy negative phenotype and von Willebrand factor. Am J Reprod Immunol.

[55] Phillips RJ, Fortier MA, Lopez Bernal A (2014). Prostaglandin pathway gene expression in human placenta, amnion and choriodecidua is differentially affected by preterm and term labour and by uterine inflammation. BMC Pregnancy Childbirth.

[56] Huang SJ, Schatz F, Masch R, Rahman M, Buchwalder L, Niven-Fairchild T, Tang C, Abrahams VM, Krikun G, Lockwood CJ (2006). Regulation of chemokine production in response to pro-inflammatory cytokines in first trimester decidual cells. J Reprod Immunol.

[57] Wolf M, Kettyle E, Sandler L, Ecker JL, Roberts J, Thadhani R (2001). Obesity and preeclampsia: the potential role of inflammation. Obstet Gynecol.

[58] Ruma M, Boggess K, Moss K, Jared H, Murtha A, Beck J, Offenbacher S (2008). Maternal periodontal disease, systemic inflammation, and risk for preeclampsia. Am J Obstet Gynecol.

[59] Genbacev O, Joslin R, Damsky CH, Polliotti BM, Fisher SJ (1996). Hypoxia alters early gestation human cytotrophoblast differentiation/invasion in vitro and models the placental defects that occur in preeclampsia. J Clin Invest.

[60] Mise H, Sagawa N, Matsumoto T, Yura S, Nanno H, Itoh H, Mori T, Masuzaki H, Hosoda K, Ogawa Y, Nakao K (1998). Augmented placental production of leptin in preeclampsia: possible involvement of placental hypoxia. J Clin Endocrinol Metab.

[61] Soleymanlou N, Jurisica I, Nevo O, Ietta F, Zhang X, Zamudio S, Post M, Caniggia I (2005). Molecular evidence of placental hypoxia in preeclampsia. J Clin Endocrinol Metab.

[62] Zhu L, Lv R, Kong L, Cheng H, Lan F, Li X (2015). Genome-wide mapping of 5mC and 5hmC identified differentially modified genomic regions in late-onset severe preeclampsia: a pilot study. PLoS One.

[63] Ching T, Ha J, Song MA, Tiirikainen M, Molnar J, Berry MJ, Towner D, Garmire LX (2015). Genome-scale hypomethylation in the cord blood DNAs associated with early onset preeclampsia. Clin Epigenetics.

[64] Liu L, Zhang X, Rong C, Rui C, Ji H, Qian YJ, Jia R, Sun L (2014). Distinct DNA methylomes of human placentas between pre-eclampsia and gestational diabetes mellitus. Cell Physiol Biochem.

[65] Xiang Y, Zhang J, Li Q, Zhou X, Wang T, Xu M, Xia S, Xing Q, Wang L, He L, Zhao X (2014). DNA methylome profiling of maternal peripheral blood and placentas reveal potential fetal DNA markers for non-invasive prenatal testing. Mol Hum Reprod.

[66] Anton L, Brown AG, Bartolomei MS, Elovitz MA (2014). Differential methylation of genes associated with cell adhesion in preeclamptic placentas. PLoS One.

[67] Ching T, Song MA, Tiirikainen M, Molnar J, Berry M, Towner D, Garmire LX (2014). Genome-wide hypermethylation coupled with promoter hypomethylation in the chorioamniotic membranes of early onset pre-eclampsia. Mol Hum Reprod.

[68] Anderson CM, Ralph JL, Wright ML, Linggi B, Ohm JE (2014). DNA methylation as a biomarker for preeclampsia. Biol Res Nurs.

[69] White WM, Brost B, Sun Z, Rose C, Craici I, Wagner SJ, Turner ST, Garovic VD (2013). Genome-wide methylation profiling demonstrates hypermethylation in maternal leukocyte DNA in preeclamptic compared to normotensive pregnancies. Hypertens Pregnancy.

[70] Blair JD, Yuen RK, Lim BK, McFadden DE, von Dadelszen P, Robinson WP (2013). Widespread DNA hypomethylation at gene enhancer regions in placentas associated with early-onset pre-eclampsia. Mol Hum Reprod.

[71] Jia RZ, Zhang X, Hu P, Liu XM, Hua XD, Wang X, Ding HJ (2012). Screening for differential methylation status in human placenta in preeclampsia using a CpG island plus promoter microarray. Int J Mol Med.

[72] van Dijk M, Visser A, Posthuma J, Poutsma A, Oudejans CB (2012). Naturally occurring variation in trophoblast invasion as a source of novel (epigenetic) biomarkers. Front Genet.

[73] Chelbi ST, Wilson ML, Veillard AC, Ingles SA, Zhang J, Mondon F, Gascoin-Lachambre G, Doridot L, Mignot TM, Rebourcet R (2012). Genetic and epigenetic mechanisms collaborate to control SERPINA3 expression and its association with placental diseases. Hum Mol Genet.

[74] Martino D, Loke YJ, Gordon L, Ollikainen M, Cruickshank MN, Saffery R, Craig JM (2013). Longitudinal, genome-scale analysis of DNA methylation in twins from birth to 18 months of age reveals rapid epigenetic change in early life and pair-specific effects of discordance. Genome Biol.

[75] Fernandez AF, Bayon GF, Urdinguio RG, Torano EG, Garcia MG, Carella A, Petrus-Reurer S, Ferrero C, Martinez-Camblor P, Cubillo I (2015). H3K4me1 marks DNA regions hypomethylated during aging in human stem and differentiated cells. Genome Res.

[76] Ollikainen M, Ismail K, Gervin K, Kyllonen A, Hakkarainen A, Lundbom J, Jarvinen EA, Harris JR, Lundbom N, Rissanen A (2015). Genome-wide blood DNA methylation alterations at regulatory elements and heterochromatic regions in monozygotic twins discordant for obesity and liver fat. Clin Epigenetics.

[77] Rushton MD, Young DA, Loughlin J, Reynard LN (2015). Differential DNA methylation and expression of inflammatory and zinc transporter genes defines subgroups of osteoarthritic hip patients. Ann Rheum Dis.

[78] Bonder MJ, Kasela S, Kals M, Tamm R, Lokk K, Barragan I, Buurman WA, Deelen P, Greve JW, Ivanov M (2014). Genetic and epigenetic regulation of gene expression in fetal and adult human livers. BMC Genomics.

[79] Simpkin AJ, Suderman M, Gaunt TR, Lyttleton O, McArdle WL, Ring SM, Tilling K, Davey Smith G, Relton CL (2015). Longitudinal analysis of DNA methylation associated with birth weight and gestational age. Hum Mol Genet.

[80] Cruickshank MN, Oshlack A, Theda C, Davis PG, Martino D, Sheehan P, Dai Y, Saffery R, Doyle LW, Craig JM (2013). Analysis of epigenetic changes in survivors of preterm birth reveals the effect of gestational age and evidence for a long term legacy. Genome Med.

[81] Raymond D, Peterson E (2011). A critical review of early-onset and late-onset preeclampsia. Obstet Gynecol Surv.

[82] Houseman EA, Accomando WP, Koestler DC, Christensen BC, Marsit CJ, Nelson HH, Wiencke JK, Kelsey KT (2012). DNA methylation arrays as surrogate measures of cell mixture distribution. BMC Bioinformatics.

[83] Hannon E, Spiers H, Viana J, Pidsley R, Burrage J, Murphy TM, Troakes C, Turecki G, O'Donovan MC, Schalkwyk LC, et al. Methylation QTLs in the developing brain and their enrichment in schizophrenia risk loci. Nat Neurosci. 2016;19:48–54. 10.1038/nn.4182 PMC4714325 26619357

[84] Kato N, Loh M, Takeuchi F, Verweij N, Wang X, Zhang W, Kelly TN, Saleheen D, Lehne B, Mateo Leach I (2015). Trans-ancestry genome-wide association study identifies 12 genetic loci influencing blood pressure and implicates a role for DNA methylation. Nat Genet.

[85] Libby G, Murphy DJ, McEwan NF, Greene SA, Forsyth JS, Chien PW, Morris AD, Collaboration DM (2007). Pre-eclampsia and the later development of type 2 diabetes in mothers and their children: an intergenerational study from the Walker cohort. Diabetologia.

[86] Sawada N, Li Y, Liao JK (2010). Novel aspects of the roles of Rac1 GTPase in the cardiovascular system. Curr Opin Pharmacol.

[87] Abraham F, Sacerdoti F, De Leon R, Gentile T, Canellada A (2012). Angiotensin II activates the calcineurin/NFAT signaling pathway and induces cyclooxygenase-2 expression in rat endometrial stromal cells. PLoS One.

[88] Shah TJ, Walsh SW (2007). Activation of NF-kappaB and expression of COX-2 in association with neutrophil infiltration in systemic vascular tissue of women with preeclampsia. Am J Obstet Gynecol.

[89] Goksu Erol AY, Nazli M, Yildiz SE (2012). Expression levels of cyclooxygenase-2, tumor necrosis factor-alpha and inducible NO synthase in placental tissue of normal and preeclamptic pregnancies. J Matern Fetal Neonatal Med.

[90] Ohgane J, Wakayama T, Senda S, Yamazaki Y, Inoue K, Ogura A, Marh J, Tanaka S, Yanagimachi R, Shiota K (2004). The Sall3 locus is an epigenetic hotspot of aberrant DNA methylation associated with placentomegaly of cloned mice. Genes Cells.

[91] Dahlstrom B, Romundstad P, Oian P, Vatten LJ, Eskild A (2008). Placenta weight in pre-eclampsia. Acta Obstet Gynecol Scand.

[92] Bilic G, Zeisberger SM, Mallik AS, Zimmermann R, Zisch AH (2008). Comparative characterization of cultured human term amnion epithelial and mesenchymal stromal cells for application in cell therapy. Cell Transplant.

[93] Thompson RF, Suzuki M, Lau KW, Greally JM (2009). A pipeline for the quantitative analysis of CG dinucleotide methylation using mass spectrometry. Bioinformatics.

[94] Borodina T, Adjaye J, Sultan M (2011). A strand-specific library preparation protocol for RNA sequencing. Methods Enzymol.

[95] Krueger F, Andrews SR (2011). Bismark: a flexible aligner and methylation caller for bisulfite-Seq applications. Bioinformatics.

[96] Li H, Handsaker B, Wysoker A, Fennell T, Ruan J, Homer N, Marth G, Abecasis G, Durbin R, Genome Project Data Processing S (2009). The sequence alignment/map format and SAMtools. Bioinformatics.

[97] Dobin A, Davis CA, Schlesinger F, Drenkow J, Zaleski C, Jha S, Batut P, Chaisson M, Gingeras TR (2013). STAR: ultrafast universal RNA-seq aligner. Bioinformatics.

[98] Anders S, Pyl PT, Huber W (2015). HTSeq—a Python framework to work with high-throughput sequencing data. Bioinformatics.

[99] Love MI, Huber W, Anders S (2014). Moderated estimation of fold change and dispersion for RNA-seq data with DESeq2. Genome Biol.

[100] Tarazona S, Garcia-Alcalde F, Dopazo J, Ferrer A, Conesa A (2011). Differential expression in RNA-seq: a matter of depth. Genome Res.

