# Supplementary material for: Amnion as a surrogate tissue reporter of the effects of maternal preeclampsia on the fetus
Source: Clin Epigenetics. 2016 Jun 10;8:67. doi: 10.1186/s13148-016-0234-1 (PMC4902972; doi:10.1186/s13148-016-0234-1)
Supplement: Additional file 1: — We described the study design, detailed analytical methods, and verification results in the supporting information file. (DOCX 21.2 MB) [file 13148_2016_234_MOESM1_ESM.docx]

**Amnion as a surrogate tissue reporter of maternal pre-eclampsia effects on the fetus.**

**Authors:**

Masako Suzuki^1^, Ryo Maekawa^1,3^, Nicole E. Patterson^1^, David M. Reynolds^2^, Brent R. Calder^1^, Sandra E. Reznik^4^, Hye J. Heo^5^, Francine F. Einstein^5^, John M. Greally^1^

**Table of content**

1. **Verification of genome-wide DNA methylation assay**
2. **Identifying cell-type specific DNA methylation**
3. **Identifying differentially methylated HpaII sites**
4. **Bisulphite MassArray analysis**
5. **Transcriptome assays**
6. **Codes used in this study**
7. **Tables**
8. **References**
9. **Verification of genome-wide DNA methylation assay**

**Micro-fluidic methylation-seq library preparation**

Verification of HELP-tagging assay was performed using single nucleotide resplution assay, sodium bisulphite sequencing. We used the Fluidigm AccessArray, a micro-fluidic amplification system to amplify the target regions according to the manufactures instructions.

**Supporting Figure 1**:

A) Flow diagram for the Microfluidic methylation assay. Genomic DNA samples were bisulphite treated and pre-amplified before the Fluidigm amplification with bisulphite PCR primer cocktail. The pre-amplified products were further microfulidically amplified, and pooled by samples, and then index sequences were added by PCR amplification. The indexed libraries were sequenced by Illumina MiSeq (250 bp paired end). B) primer design principles. Since the massively parallel sequencing reads the primer sequence at first, and the bisulphite forward primers were G-rich and reverse primers were C-rich, we designed primers to sequence evenly sequenced from both side to avoid GC% skew.

In **Supporting Figure 1**, we illustrated the assay workflow and primer design principles. We treated 500 ng of genomic DNA using Zymo DNA methylation gold kit to convert unmethylated cytosines to uracils. To increase amplifiable templates per chamber, we pre-amplified bisulphite treated DNA with pooled gene specific primers (25 µl of 2x KAPA HiFi HotStart Uracil+ ReadyMix, 5 µl of 0.002 µM of pooled primers, and 250 ng of bisulphite treated DNA in a final volume of 50 µl; 95 ^o^C for 1 minutes, then 15 cycles of 98 ^o^C for 30 seconds, 60 ^o^C for 30 seconds and 72 ^o^C for 30 seconds followed by 10 minutes at 72 ^o^C for final extension). We estimated at least 100 copies of amplifiable template per chamber. The primer sequences we used this studies were listed **Supporting** **Table 1**. After purifying the pre-amplified products in a final volume of 15 µl using AMPure XP beads (1:0.8 dilution), Micro-fluidic amplification was performed with Fluidigm AccessArray (IFC Controller, FC1 Cycler and Access Array IFC) according to the manufacturer’s protocol, except we used KAPA HiFi HotStart Uracil+ ReadyMix (KAPA Byosystems) to amplify the libraries (35 cycles, 4 µl of mixture of 4 µl of 2x KAPA HiFi HotStart Uracil+ ReadyMix and 1 µl of purified products was applied into each of the sample inlets, 4 µl of 4 µM each of gene specific forward and reverse primers in each of the primer inlets). After the micro-fluidic amplification, the amplified products were pooled by sample and purified using AMPure XP beads (1:0.8 dilution), then added adapter sequences using further PCR amplification for the Illumina sequencer (10 cycles, 10 µl of 2x KAPA HiFi HotStart Uracil+ ReadyMix, 2 µl of 0.4 µM of adapter-added primers, 1 µl of amplified products in a final volume of 20 µl). We tested the library quality with bioanalyzer (**Supporting Figure 2**) and quantified the concentration with Qubit fluorometer. After the quality check, we ran the quality passed libraries on Illumina MiSeq sequencer.

**Supporting Figure 2:**

Bioanalyzer traces of before and after adding Illumina sequencing adapters. As we expected, we observed size shift (~60 bp increase) after adding Illumina sequencing adapters. Control DNA : HCT 116 Dnmt TKO DNA (Zymo Research).

**Calculating DNA methylation percentage from Micro-fluidic methylation-seq results**

To eliminate optical duplicated reads, we eliminated reads less than 1000 pixel apart each other using *Picard MarkDuplicates* function. Obtained sequences were trimmed off the adapter sequences using trim_galore (ver 0.3.3) (http://www.bioinformatics.babraham.ac.uk/projects/trim_galore/), and then aligned to bisulphite converted genomic sequences of targeted regions (hg19) with *bismark* (ver 0.10.1) [1]. DNA methylation percentage was calculated as proportion of observed unconverted cytosine number in the total number of observed cytosine at the CpG site. We selected CpG sites which all samples have at least 20x coverage. Each percentage of methylation was listed in **Supporting Table 2**.

**Testing correlation between Micro-fluidic methylation-seq and HELP-tagging**

First, we tested the microfluidic methylation seq with commercially available fully methylated and unmethylated human genomic DNA (both from Zymo Research). To test the linearly of the results, we mixed fully methylated and unmethylated DNA as 0%, 25%, 50% and 100% methylated). We tested 27 assays in total and the regression formula from average slope and intersect was y= 0.98*x +1.78 with y indicating observed methylation and x indicating sample mixture rate. We compared between the methylation values of Micro-fluidic methylation-seq and HELP-tagging angle values with spearman and person correlation (**Supporting Figure 3B**). The interquartile range of correlation correlation values were -0.75 to -0.84 (pearson, mean -0.78) and -0.70 to -0.80 (spearman, median -0.75).

**Supporting Figure 3**:

A) Observed %methylation and expected %methylation from control DNA. We mixed commercially available non-methylated control DNA (HCT116 Dnmt1 and Dnmt3b double knockout (HCT116 DKO)) and methylated control (enzymatically methylated HCT116 DKO) (both from Zymo Research). The observed %methylation was calculated from massively parallel sequencing results using BSMap. The results showed a clear positive correlation between observed %methylation and expected %methylation in control DNA.

B) Comparison between the DNA methylation values of Microfluidic methylation-seq and HELP-tagging.

1. **Identifying cell-type specific DNA methylation**

**Amniotic epithelial and stromal cell isolation**

To obtain cell-type specific DNA methylation, we selectively collected amniotic epithelial cells and stromal cells from amnion membrane [2]. Amnion membranes were collected immediately after the delivery and washed with pen-strep supplemented DMEM (Invitrogen). Then, we performed serial digestions on the amnion membranes. All incubations were performed at 37^o^C in a 5% CO_2_ incubator. First, we treated membranes with 0.25% trypsin for 3 minutes to eliminate cellular debris and blood contamination, then 1.2 units/ml of dispase for 60 minutes followed by gentle manual scraping of the amnion epithelial surface with a cell scraper to detach the epithelial layer from the membrane. After tested the complete removal of epithelial cells under the microscope, the membrane was further digested with 2 mg/ml collagenease A for 90 minutes to complete digest for stromal cell collection. Collected epithelial and stromal cells were filtered with cell strainer and half of cells were snap-frozen (no culture) and other half was cultured in a 1:1 mixture of Ham’s F-12 and Dulbecco’s modified Eagle’s medium supplemented with 10% fetal bovine serum (FBS), 100 units/ml penicillin and 100 µg/ml streptomycin. Cell passage was performed using trypsin-EDTA at 80% of confluent. We used three pairs of snap-frozen samples and two pairs of cultured cells (P1). In **Supporting Figure 4**, we showed images of cultured AS and AE.

Supporting Figure 4: cellular morphology of amniotic epithelial cells rich and stromal cell rich fractions.

**Finding cell-type specifically methylated HpaII sites using low coverage whole genome bisulphite sequencing**

To find cell-type specific DNA methylation, we performed low coverage bisulphite sequencing on 5 pairs of epithelial and stromal cells from same individual. Genomic DNA was extracted using phenol-chloroform treatment method with ethanol precipitation. Extracted genomic DNA was fragmented (300-400 bp) with Covaris, end-repaired, dA tailed and then premethylated adapters (Illumina TruSeq adapters) were ligated at the ends of the fragmented DNA. The adapter ligated DNA samples were purified with AMPure XP beads (1:1 dilution) to eliminate adapter dimers and products with short insert, and then treated with sodium bisulphite using EZ DNA Methylation Gold kit (Zymo Research). The bisulphite treated products were used as a template for PCR amplification using following condition: 25 µl of 2x KAPA HiFi HotStart Uracil+ ReadyMix, 1.5 µl of 10 µM of primer P5, 1.5 µl of 10 µM of primer P7 and bisulphite treated library in a final volume of 50 µl; 98^o^C for 2 minutes, then 10 cycles of 98 ^o^C for 30 seconds, 60 ^o^C for 30 seconds and 72 ^o^C for 4 minutes followed by 10 minutes at 72 ^o^C for final extension. Amplified libraries were purified with AMPure XP beads (1:1 dilution) and then pooled libraries were sequenced using Illumina HiSeq2500 (100 bp paired-end reads). The obtained sequences were trimmed of the adapter sequences, and aligned to bisulphite converted human reference genome (hg19) using *Bismark* (version 0.13.0). We summarized the sequence results on **Supporting Table 3**. We selected significantly differentially methylated HpaII site with the average coverage of all 10 samples was greater than 3 (p<0.0001, Fisher’s exact test) as candidate. We tested the DNA methylation status with single nucleotide resolution DNA methylation assays (bisulphite direct sequencing and bisulphite MassArray). We confirmed three HpaII sites (Hpa_1553647, Hpa_210409 and Hpa_621984) were differentially methylated between epithelial and stromal cells (**Figure S1**). The primers we used were listed in **Supporting Table 4**.

1. **Identifying differentially methylated HpaII sites**

We illustrated an analytical workflow in **Supporting** **Figure 5**. After adjusting for batches using Combat in SVA package, we eliminated the HpaII sites which tag-fragment (-28 to +28 from the second C of HpaII sites) is overlapped with known SNP (dbSNP142). In addition, we eliminated HpaII site which located in RepeatMasker Annotation tracks and MspI reference read number smaller than 4. Then, we eliminated HpaII sites which have strong correlation to HpaII_1553647 (r>0.3, spearman correlation) which was identified as a stromal-epithelial cell type specific methylation site. After eliminating all potential confounder affected HpaII sites, we had 545,961 HpaII sites to test. Using this high-confidence data set, we tested the degree of contribution of known clinical covariates to DNA methylation profiles using principal components analysis (PCA, **Figure1 and Supporting Figure 6**). To identify the DNA methylation changes, we ran the following three regression models and extracted the proportion of variance explained from each:

**Supporting Figure 5:**

An analytical workflow for identifying differentially methylated HpaII sites.

Model 1: HpaII methylation ~ Maximum systolic blood pressure (continuous)

Model 2: HpaII methylation ~ Proteinuria grade (categorical)

Model 3: HpaII methylation ~ Proteinuria grade (continuous)+ Maximum systolic blood pressure (categorical)

**Supporting Figure 6:**

Bar plots showed the ranking of significance of each covariate to each principal components (PC1 to PC5). The p-values were calculated with the linear regressions of top five principal components onto each known covariates.

We tested the significance with ANOVA and selected HpaII sites which the model had p<0.05 (FDR adjusted) and median difference between groups were greater than 10. We listed the differentially methylate HpaII sites of each model in **Table 3S**. To find variable HpaII sites, we compared the variances of each HpaII sites in controls and PE-exposed fetuses with the F-test (R project, *stats* package) and selected variable HpaII (var-HpaII) sites where the FDR adjusted p-value < 10^-7^ and the ratio of variance >20.

1. **Bisulphite MassArray analysis**

To confirm the DNA methylation changes, we designed primer sets to test the methylation status with bisulphite treatment based approach.

Primers were designed with MethPrimer (<http://www.urogene.org/methprimer/>) as following setting;

Product size (bp): Min 100, opt 250, Max 400

Primer Tm (^o^C): Min 54, Opt 58, Max 60

Primer size (bp): Min 23, Opt 25, Max 29

Minimum product CpG number: 1

Maximum poly X number in primer: 5

Maximum non-CpG ‘C’ number in primer: 4

Maximum poly T number in primer: 8

All primers were tested the amplification specificity with BiSearch ePCR function (<http://bisearch.enzim.hu/?m=genompsearch>) and the analyzability with the amplicon prediction algorithm, a part of Bioconductor package MassArray (<http://www.bioconductor.org/packages/release/bioc/html/MassArray.html>) [ref]. For Bisulphite MassArray with EpiTYPER (SEQUENOME), we added tag sequences at the 5’ end of both forward (5’- aggaagagag- 3’) and reverse (5’- cagtaatacgactcactatagggagaaggct -3’) primers. We used 500 ng of DNA for sodium bisulphite treatment using EZ DNA methylation Gold kit (Zymo Research) according to the manufacture’s instructions. To amplify the products, we used FastStart High Fidelity PCR System (Sigma-Aldrich) with following condition;

Mixture (final volume 25 µl):

10x PCR buffer with 18 mM MgCl_2_ 2.5 µl

10 µM primer (forward) 1 µl

10 µM primer (reverse) 1 µl

Template (correspond to 10 ng of original material) 1 µl

Polymerase (5 units/µl) 0.25 µl

PCR grade water 19.25 µl

PCR condition:

95 ^o^C 10 minutes

42 cycles of

95 ^o^C 30 seconds

Primer Tm 30 seconds

72 ^o^C 30 seconds

72 ^o^C 10 minutes

4 ^o^C hold

After the amplification, we checked the products on 2% agarose gel with GelRed staining (Biotium, Inc.), then the products were analyzed the DNA methylation percentage using EpiTYPER (AGENA/SEQUENOM). The results of DNA methylation percentage were listed in **Supporting Table 5** and the primers we used were listed in **Supporting Table 4**.

1. **Transcriptome assays**

We tested the transcription status of 32 amnion sample (17 PE and 12 Controls) with directional RNAseq method. Total RNA was extracted using TRIzol reagent from amnion membrane and the total RNA was then treated with DNaseI to eliminate residual DNA. The 500 ng of extracted RNA samples were subjected to directional RNA seq library preparation. The protocol we used consists of a combination of reverse transcription of ribosomal-RNA-depleted RNA and second-strand synthesis with dUTP using the approach of Parkhomchuck *et al*. [3]. Using ribosomal-RNA-depleted RNA allows us to use a mixture of oligo-d(T) and random hexamers as RT primers, the dUTP incorporation at the second-strand cDNA synthesis maintains the direction of transcript. All libraries were sequenced by Illumina HiSeq2500, and we used gsnap (ver 2012-05-07) [4] for the alignment and htseq (v0.5.3p3) [5] for counting transcripts. The alignment and counting transcripts were performed using automated WASP pipeline (WASP-3.1.3 (ref. 6589)) [6]. After the htseq counting, the counts were normalized using DEseq2 [7]. To find the differentially expressed genes, we applied the normalized counts for NOIseq algorism [8]. We validated the expression status using quantitative RT-PCR (**Supporting Figure 7**). The primers we used this study are listed in **Supporting Table 4**.

**Supporting Figure 7:**

Verification of directional RNA-seq results with quantitative RT-PCR.

1. **Codes used in this study**

The codes used in this study were listed on code.txt and were distributed on https://github.com/GreallyLab/PE_Suzuki_et_al_2016

1. **Tables**

Supporting Table 1: Oligo sequences of Micro-fluidic methylation seq analysis

Supporting Table 2: Micro-fluidic methylation seq results

Supporting Table 3: low coverage bisulphite sequencing summary

Supporting Table 4: Lists of primers used in this study

Supporting Table 5: Bisulphite MassArray results

Supporting Table 6: A list of correlation values between epithelial cell amount and methylation

All supporting tables were stored in the Supporting tables.xlsx.

1. **References**

1. Krueger F, Andrews SR. Bismark: a flexible aligner and methylation caller for Bisulfite-Seq applications. Bioinformatics. 2011;27(11):1571-2. doi: 10.1093/bioinformatics/btr167. PubMed PMID: 21493656; PubMed Central PMCID: PMC3102221.

2. Bilic G, Zeisberger SM, Mallik AS, Zimmermann R, Zisch AH. Comparative characterization of cultured human term amnion epithelial and mesenchymal stromal cells for application in cell therapy. Cell transplantation. 2008;17(8):955-68. PubMed PMID: 19069637.

3. Parkhomchuk D, Borodina T, Amstislavskiy V, Banaru M, Hallen L, Krobitsch S, et al. Transcriptome analysis by strand-specific sequencing of complementary DNA. Nucleic Acids Res. 2009;37(18):e123. Epub 2009/07/22. doi: gkp596 [pii]

10.1093/nar/gkp596. PubMed PMID: 19620212; PubMed Central PMCID: PMC2764448.

4. Wu TD, Nacu S. Fast and SNP-tolerant detection of complex variants and splicing in short reads. Bioinformatics. 2010;26(7):873-81. Epub 2010/02/12. doi: btq057 [pii]

10.1093/bioinformatics/btq057. PubMed PMID: 20147302; PubMed Central PMCID: PMC2844994.

5. Anders S, Pyl PT, Huber W. HTSeq--a Python framework to work with high-throughput sequencing data. Bioinformatics. 2015;31(2):166-9. doi: 10.1093/bioinformatics/btu638. PubMed PMID: 25260700; PubMed Central PMCID: PMCPMC4287950.

6. McLellan AS, Dubin RA, Jing Q, Broin PO, Moskowitz D, Suzuki M, et al. The Wasp System: an open source environment for managing and analyzing genomic data. Genomics. 2012;100(6):345-51. doi: 10.1016/j.ygeno.2012.08.005. PubMed PMID: 22944616.

7. Love MI, Huber W, Anders S. Moderated estimation of fold change and dispersion for RNA-seq data with DESeq2. Genome Biol. 2014;15(12):550. doi: 10.1186/s13059-014-0550-8. PubMed PMID: 25516281; PubMed Central PMCID: PMCPMC4302049.

8. Tarazona S, Garcia-Alcalde F, Dopazo J, Ferrer A, Conesa A. Differential expression in RNA-seq: a matter of depth. Genome Res. 2011;21(12):2213-23. doi: 10.1101/gr.124321.111. PubMed PMID: 21903743; PubMed Central PMCID: PMC3227109.
