# Supplementary figures and images for: Amnion as a surrogate tissue reporter of the effects of maternal preeclampsia on the fetus
Source: Clin Epigenetics. 2016 Jun 10;8:67. doi: 10.1186/s13148-016-0234-1 (PMC4902972; doi:10.1186/s13148-016-0234-1)

A

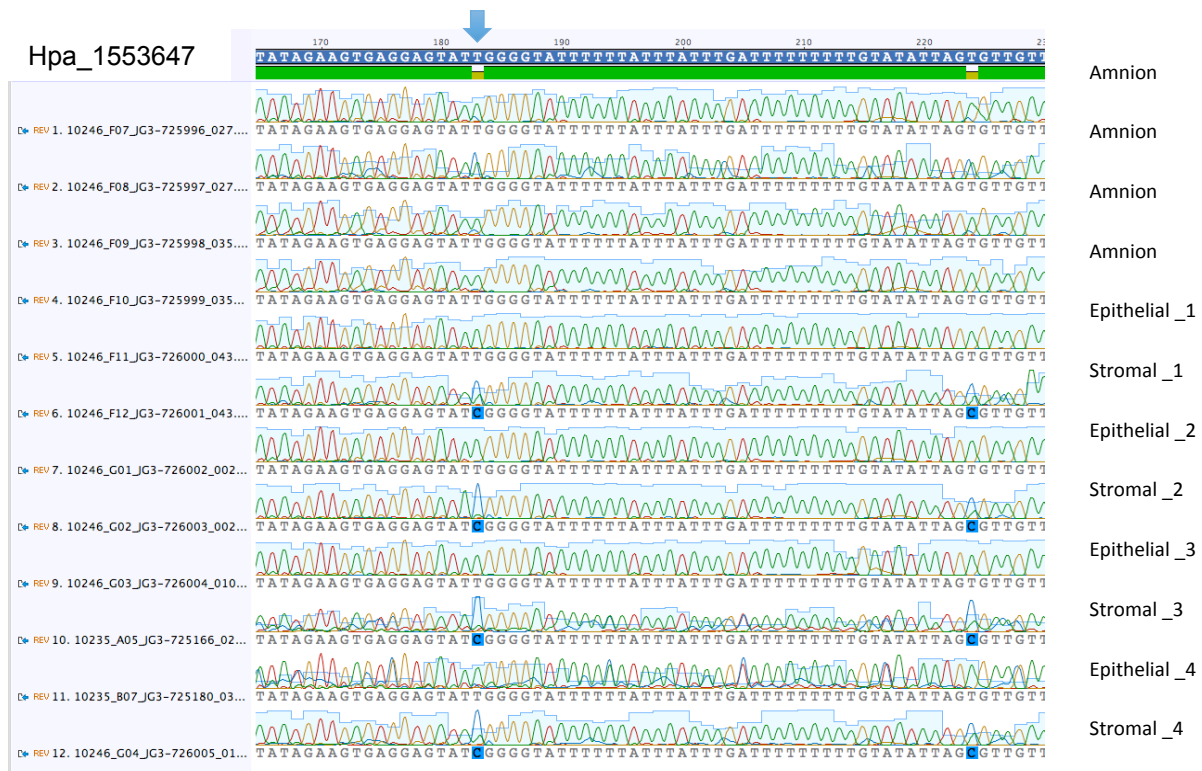

B

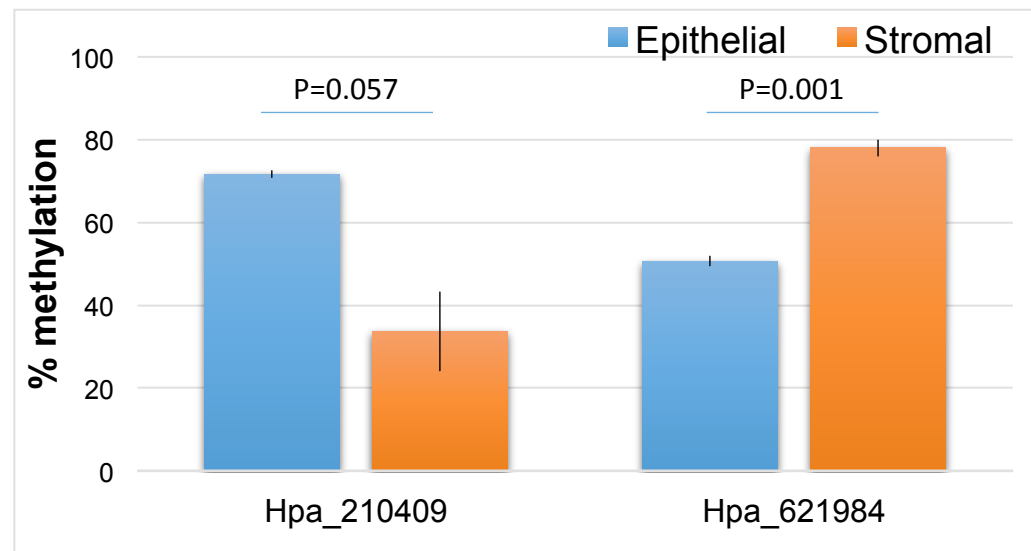

Figure S1

Supplement: Additional file 2: Figure S1. — Cell type-specific DNA methylation. A) Bisulfite sequencing results of Hpa_1553647. Each row represents the sequence result from bisulfite PCR products. We show four amnion, four amniotic epithelial cell, and amniotic stromal cell results. The amniotic epithelial and stromal cells were isolated from four individuals. The blue arrow indicates the Hpa_1553647 position in the sequencing results. B) Bisulfite MassArray results of Hpa_210409 and Hpa_621984. The y-axis shows the % DNA methylation in amniotic epithelial and amniotic stromal cells (from four individuals). The p values were calculated by a t test. The error bars indicate the standard deviations. (PDF 317 kb) [file 13148_2016_234_MOESM2_ESM.pdf]

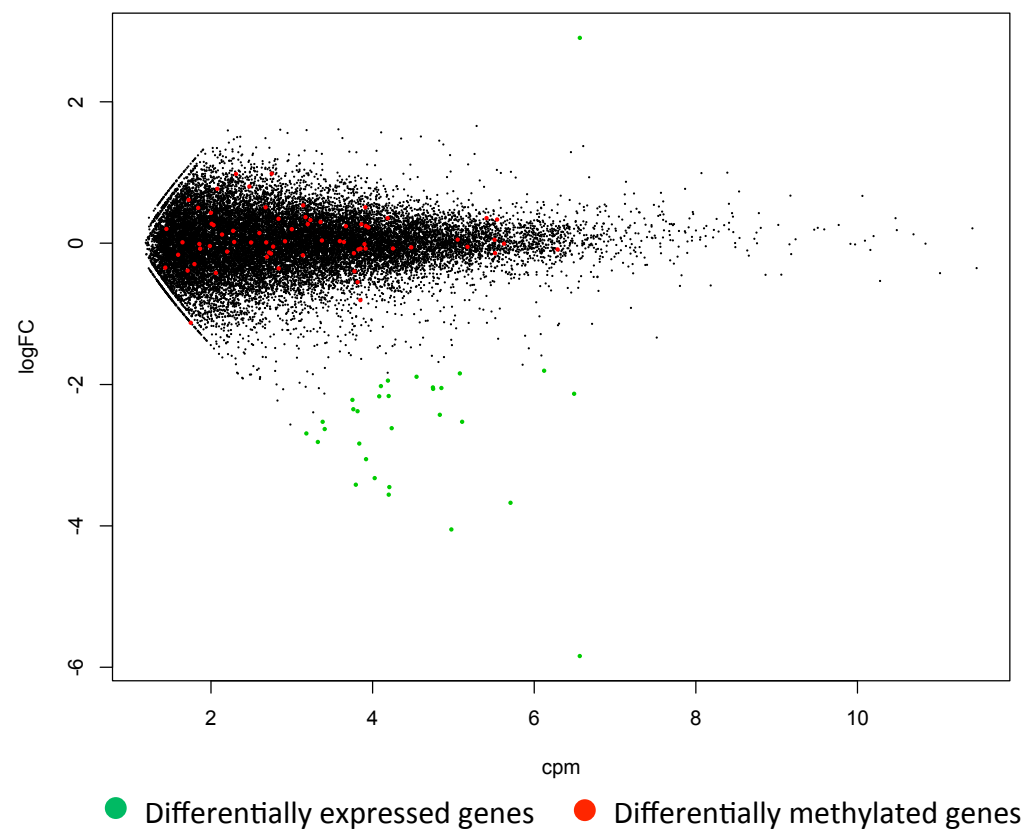

Figure S2

Supplement: Additional file 4: Figure S2. — Differentially expressed genes in preeclampsia-exposed amnion. A MA plot shows the distribution of differentially expressed and differentially methylated genes. The x-axis shows counts per million (CPM), and the y-axis shows log-fold changes. The differentially methylated genes are highlighted in red and the differentially expressed genes are highlighted in green. (PDF 4.30 MB) [file 13148_2016_234_MOESM4_ESM.pdf]

SALL3

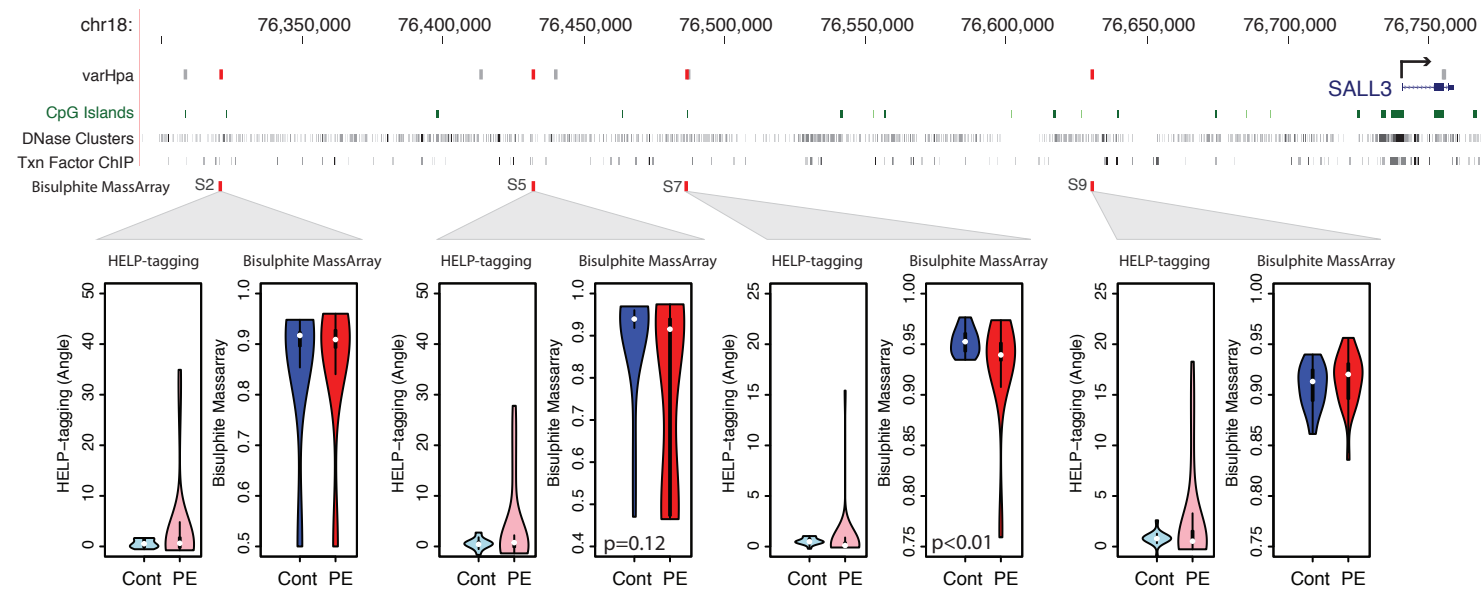

KCNMA1

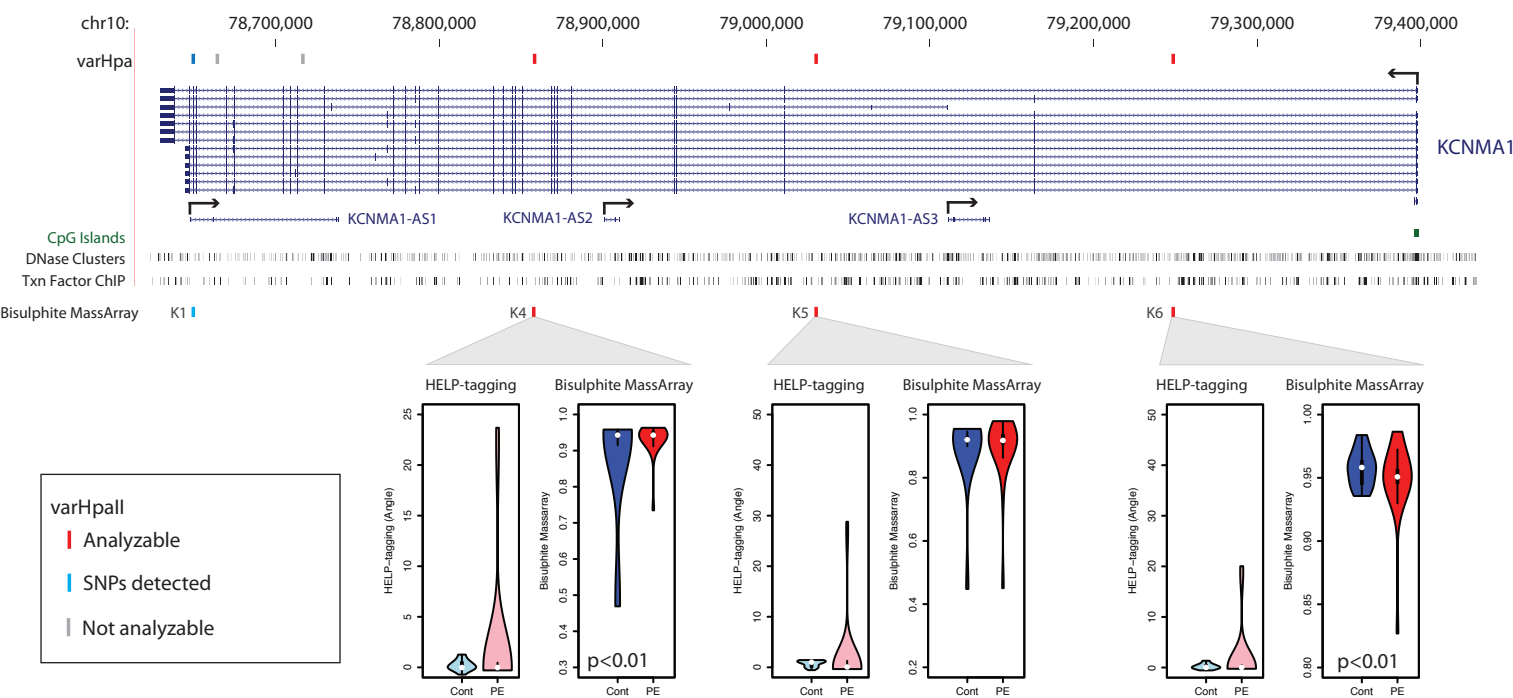

Supplement: Additional file 5: Figure S3. — Genes with local variable HpaII sites. HpaII sites with variable DNA methylation are shown at specific loci with HELP-tagging (angle values = (1 − DNA methylation value)) and corresponding bisulfite MassArray verification results. The var-HpaII track indicates loci that could be analyzed by bisulfite MassArray in red, and those that could not be analyzed in gray (PCR amplification problem or more than 2 CpG sites in a MassArray fragment). The blue loci are those in which the analysis of the amplicon indicated the likely presence of a sequence polymorphism [93]. Top panel: SALL3 region, bottom panel: KCNMA1 region. (PDF 1.33 MB) [file 13148_2016_234_MOESM5_ESM.pdf]

Bisulphite MassArray

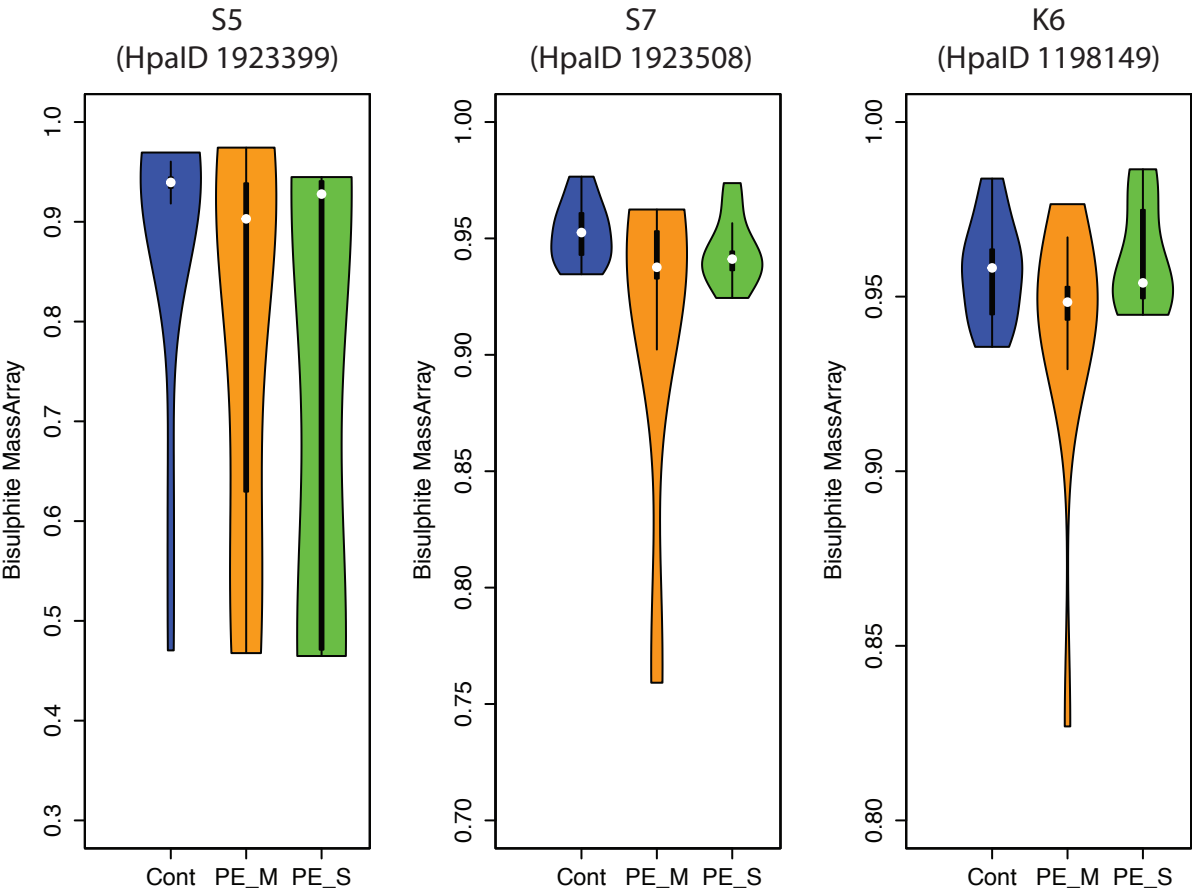

Supplement: Additional file 6: Figure S4. — Comparison of the DNA methylation distribution of variable HpaII sites. The distributions of the DNA methylation levels of variable methylation sites in severe PE-exposed (PE_S, green) (proteinuria grade ≥3 and systolic blood pressure ≥160 mmHg), less severe PE-exposed (PE_M, orange) (proteinuria grade ≤1 and systolic blood pressure ≥ 140 mmHg) and control (blue) were summarized graphically in violin plots. (PDF 895 kb) [file 13148_2016_234_MOESM6_ESM.pdf]
